# Screening and determinant of suspected developmental delays among Egyptian preschool-aged children: a cross-sectional national community-based study

**DOI:** 10.1186/s12887-023-04335-0

**Published:** 2023-10-19

**Authors:** Ammal M. Metwally, Ali M. Abdallah, Ebtissam M. Salah El-Din, Dina Abu Zeid, Zeinab Khadr, Ghada A. Elshaarawy, Alshaimaa A. Elkhatib, Amal Elsaied, Engy A. Ashaat, Nahed A. Elghareeb, Mohamed H. Abdou, Asmaa M. Fathy, Sherif E. Eldeeb, Mohamed AbdAllah, Muhammed Al-tohamy Soliman, Rokia AbdElshafy S. El Banna, Abdelrahman K. Hassanein, Thanaa M. Rabah, Mohamed Abdelrahman, Sara F. Sallam

**Affiliations:** 1 Community Medicine Research Department/ Medical Research and Clinical Studies Institute, National Research Centre (Affiliation ID: 60014618), Public Health and Community Medicine, Dokki, P.O. 12622, Giza, Egypt; 2 Quantitative Methods Department - Aswan University, Aswan, Egypt; 3 Child Health Department/ Medical Research and Clinical Studies Institute, National Research Centre (Affiliation ID: 60014618), Dokki, Giza, Egypt; 4 Department of Statistics, Faculty of Economics and Political Science, Cairo University, Cairo, Egypt; 5 The Social Research Center of the American University in Cairo, Cairo, Egypt; 6 Child With Special Needs Department/ Medical Research and Clinical Studies Institute, National Research Centre (Affiliation ID: 60014618), Dokki, Giza, Egypt; 7 Clinical Genetics Department/ Human Genetics and Genome Research Institute, National Research Centre (Affiliation ID: 60014618), Dokki, Giza, Egypt; 8 Prevention of Disability General Directorate, Ministry of Health and Population, Cairo, Egypt; 9 Mansoura Health Directorate, Ministry of Health and Population, Mansoura, Dakahlia Egypt; 10 Complementary Medicine Department/ Medical Research and Clinical Studies Institute/National Research Centre (Affiliation ID: 60014618), Dokki, Giza, Egypt; 11 Biological Anthropology Department/ Medical Research and Clinical Studies Institute/National Research Centre (Affiliation ID: 60014618), Dokki, Giza, Egypt

**Keywords:** Preschool children, Developmental delay, Prevalence, Milestones, Risk factors, Language delay, Fine motor, Gross motor, Personal-social, Screening

## Abstract

**Background:**

Early childhood life is critical for optimal development and is the foundation of future well-being. Genetic, sociocultural, and environmental factors are important determinants of child development.

**Aim:**

The objectives were to screen for suspected developmental delays (DDs) among Egyptian preschool children, and to explore the determinants of these delays based on sociodemographic, epidemiological, maternal, and child perinatal risk factors.

**Methods:**

A national Egyptian cross-sectional developmental screening of a representative sample of preschool children (21,316 children) aged 12 to 71 months. The Revised Denver Prescreening Developmental Questionnaire (R-PDQ) followed by the Denver Developmental Screening Test, 2^nd^ edition (DDST) was used.

**Results:**

Each screened child manifested at least one of six developmental categories. Either typical development, gross motor delay (GM), fine motor adaptive delay (FMA), Language delay (L), Personal-social delay (PS), or multiple DDs. The prevalence of preschool children with at least one DD was 6.4%, while 4.5% had multiple DDs. Developmental language delay was the most prevalent, affecting 4.2% of children. The least affected domain was GM (1.9% of children). Boys were more likely to have DD than girls. Children in urban communities were more likely to have at least one DD than those in rural areas (OR = 1.28, 95%CI: 1.14–1.42), and children of middle social class than of low or high social class (OR = 1.49, 95%CI: 1.30–1.70 & OR = 1.40, 95%CI: 1.23–1.59 respectively). The strong perinatal predictors for at least one DD were children with a history of postnatal convulsions (OR = 2.68, 95%CI: 1.97–3.64), low birth weight (OR = 2.06, 95%CI: 1.69–2.52), or history of postnatal cyanosis (OR = 1.77, 95%CI: 1.26–2.49) and mothers had any health problem during pregnancy (OR = 1.73, 95%CI: 1.44–2.07). Higher paternal and maternal education decreased the odds of having any DD by 43% (OR = 0.57, 95% CI: 0.47–0.68) and 31% (OR = 0.69, 95%CI: 0.58–0.82) respectively.

**Conclusion:**

This study demonstrates a considerable attempt to assess the types and the prevalence of DD among preschool children in Egypt. Perinatal factors are among the most common determinants of DD in preschool children and the majority could be preventable risk factors.

**Supplementary Information:**

The online version contains supplementary material available at 10.1186/s12887-023-04335-0.

## Introduction

A child who doesn’t achieve normal developmental milestones at the expected age is described to have developmental delay (DD) [1]. During the first 5 years of life, the fastest brain growth and development occur. A lot of nutritional, physical, psychological, and social variables are documented to affect children in all aspects of development throughout the whole childhood period and even before childbirth [2–5].

Developmental delay can be in single (i.e., isolated) domain or more of the main well-known developmental domains which are gross motor, fine motor, adaptive- behavioral, language, cognitive, and social-emotional skills [6, 7]. If a delay occurs in 2 or more of these domains, this child is identified to have global developmental delay (GDD) [8]. Gross motor skills are seen in jumping, catching, kicking, and stability. Meanwhile, fine motor skills are seen in holding a pencil, opening a lunch box, dressing, etc. Both skills are under the Motor development domain which is one of the key domains of early learning and development that affects school short and long-term academic success [9]. Whereas, developmental Language delay is the most common preschool developmental delay [10]. Any language delay will interfere with children’s abilities in learning and understanding vocabulary. Grammar, inferring meaning, and expressive language are also affected by such delay. All these language-related abilities are important for children as they are linked to cognitive abilities, self-perception as well as sport-specific skills [11–13]. Personal-Social and Emotional Development supports children to have a confident sense of themselves, respect for others, social skills, emotional well-being, and a positive outlook on learning. These are all crucial for school readiness and developing confidence and independence [14–16].

Five to ten percent of the pediatric population has been estimated to experience developmental delays [6]. The prevalence of developmental delay among preschool children had a wide range according to the used assessment tool, it ranges from 1.5% to 19.8% in different studies [17–21]. In Egypt, a previously reported prevalence was 3.4- 3.6% [22], displaying the lack of a representative study on which national intervention plans can be prepared and implemented. The most widely used report suggested that 250 million children are at risk of developing suboptimal development due to extreme poverty or stunting and disregarding other risk factors [6]. Early identification of developmental delays and their risk factors is important for appropriate management that can positively alter the child’s developmental trajectory [12, 13, 23].

The American Academy of Pediatrics recommended the administration of standardized screening tools at the ages (9, 18, 24, or 30 months) to produce effective developmental surveillance [24]. Furthermore, the Sustainable Development Goals (SDGs) highlighted the importance of monitoring children's health and development as children with DDs and disabilities are at greater risk of suboptimal health, educational attainment, and well-being than children without such disabilities [21]. Accordingly, the aim of the present study is to screen for suspected developmental delays (DDs) among Egyptian preschool children; including gross motor delay (GM), fine motor delay (FM), language (L) delay, and delay of personal-social (PS) skills. The study explored the risk factors for DDs, based on the socio-demographic, epidemiological, maternal, and child health factors for provision of effective surveillance and prevention of childhood disabilities.

## Materials and methods

### Study design and setting

A Cross-sectional national survey was conducted during a period of two years starting from December 2017 till December 2019. This community-based survey was directed to randomly selected households from 8 Governorates representing the four geographic regions of Egypt: *urban cities (Cairo)*, *rural and urban Upper Egypt (Fayoum, Assuit, and Aswan), rural and urban Lower Egypt (Damietta, Dakahlia, and Gharbia)* and *Frontier governorates (Marsa Matrouh*). Egypt has 27 governorates, some are a mix of urban and rural, while other governates are just cities. The choice of the number of governorates was according to their population density according to the enumeration census from the Central Agency for Public Mobilization and Statistics (CAPMAS) [25].

### Target group

The study targeted parents or caregivers of all children aged 12 – 71 months (< 6 years) at the visited houses.

#### Inclusion and exclusion criteria

In fact, this study aimed to screen for suspected developmental delay in early childhood in order to direct the attention of parents and instructors in early childhood programs towards children who need extra efforts or special programs. Accordingly, children who were designated by parents as typically developed (experienced normal milestones for their ages) or with atypical development were included in the current study. The child was included in the study if he/ she had not diagnosed before with developmental disability, but parents have noticed slowness in one or more of the areas of development (Gross motor, fine motor-adaptive, Language, and personal-social). The slowness to reach milestones for a child's age in one or more of the areas of development is considered a developmental delay (DD) [26]. The affected milestones were categorized into one of four domains: Gross motor, fine motor-adaptive, Language, and personal-social.

*Exclusion criteria* were: Children with genetic disorders (e.g., Turner syndrome, Down syndrome, or fragile-X syndrome) who had positive genetic test results, children with movement disorders due to isolated orthopedic problems, congenital deformities of limbs, or children with disabilities affecting thinking, learning, or social relationships (e.g., Children with intellectual disabilities or with autism spectrum disorder who had been diagnosed by previous psychological testing) [27]. The previously mentioned disorders were excluded from the survey following history taking and Looking at positive reports from specialist of the Ministry of Health and Population (MOHP) or private clinics. The professional clinical genetic team from the National Research Centre was concerned with the confirmation of suspicious genetic disorders characterized by definite physical features. The research team referred all children with disabilities to specialized centers belonging to MOHP for further confirmation, management and rehabilitation. Children with possible affection of vision or hearing who had no diagnostic reports, were referred for specific centers for formal vision or hearing assessment.

As this manuscript is a piece of work that is derived from a mega project titled “National Prevalence Survey for Autism Spectrum Disorders (ASDs): Assessing its Epidemiological Pattern and Risk Factors”, so the children already diagnosed with developmental disabilities [28], and autism [29], were published in another two manuscripts, and the third one is under publication. This could justify the exclusion of children with diagnosed developmental problems.

### Sampling frame and cluster preparation

**Fig. 1:**
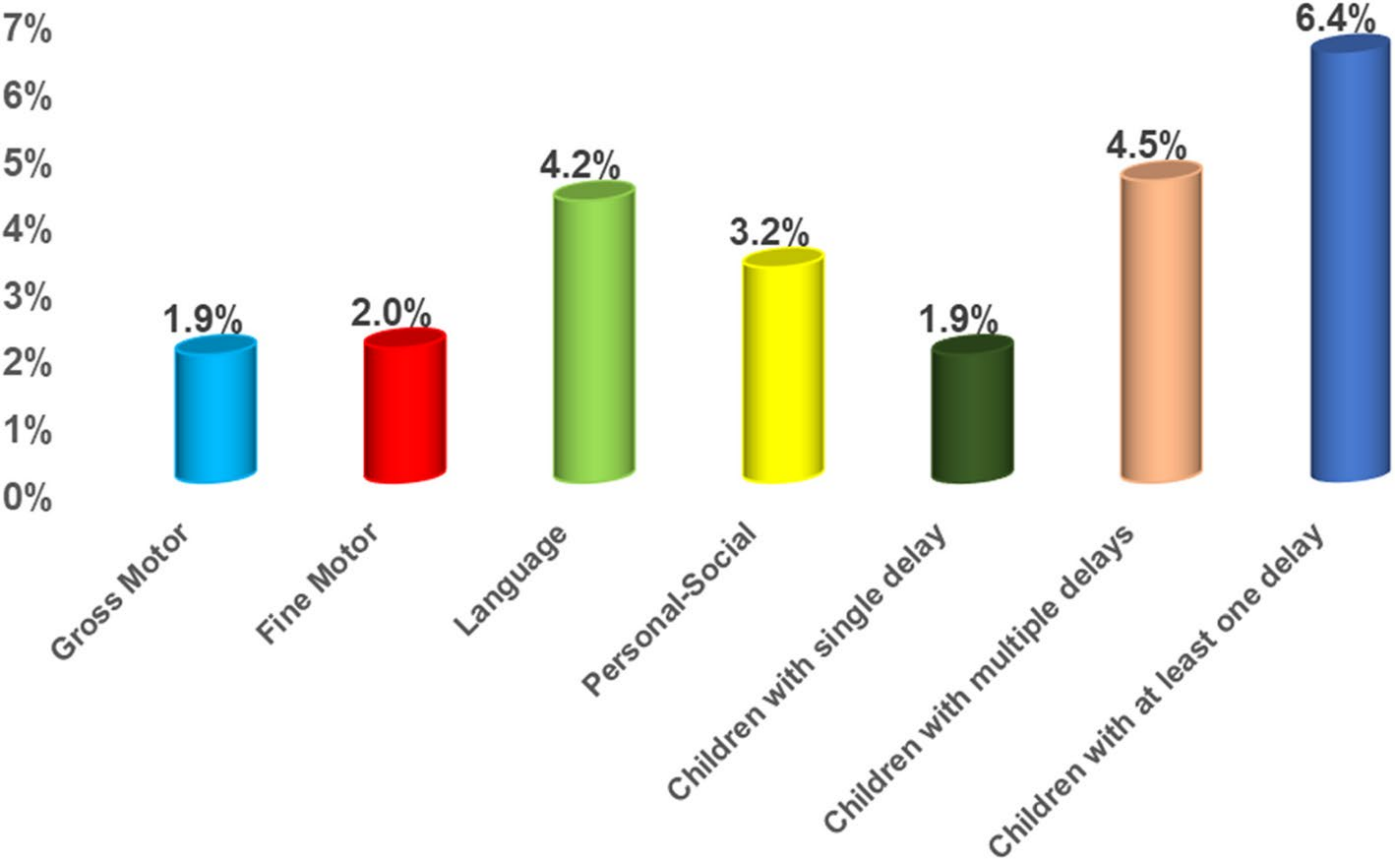
Prevalence of the studied types of delays among children aged 1- < 6 years (Surveyed children: 21,316)

A multistage cluster random sampling technique was used with three sampling frames in three stages; *the first sampling frame* used was the comprehensive list of the 27 governorates in Egypt. In the first stage, a representative sample of 8 governorates was selected to be proportionate to the population size representing the main geographic areas in Egypt as mentioned before under the title of study design and as shown in S-Fig. 1: which represent Map of the 27 Egyptian governorates distributed within the four geographic regions (adapted using data from the Humanitarian Data Exchange under the CC BY-IGO license [30].

*The second frame* used was the choice of a representative sample of districts and local units from each governorate taking into consideration the differences in the socioeconomic levels, using the socioeconomic status scale for health research in Egypt [31] Accordingly, three social categories; namely low, medium and high social classes were identified. Three districts were selected per each of the Urban and Rural localities with one representing one social class. *The third frame followed the same randomization pattern*. A total of 45-blocks was selected with 3 blocks from Cairo and six blocks from each of the other governorates. Such stratification ensured both the adequate sample size and heterogenicity of the data collected. In this stage, households in the selected city village blocks were screened.

### Survey sample size

A sample size of 21,392 children aged 1- 6 years produces a two-sided 98% confidence interval (confidence limit) with a width equal to 0.010 (margin of error) when the sample proportion is 0.120 [32]. The sample was distributed according to the Central Agency for Public Mobilization and Statistics (CAPMS) census in 2017 [25]. Sample size calculation is based on the estimated prevalence of each domain of developmental delays with 1 to 10% of the pediatric population [7]. The least prevalence was taken into consideration to ensure the largest accurate and representative national estimatesof DDs. Cases with completed questionnaires amounted to 21,316 with a 0.4% loss rate.

### Study instruments

The mother or the caregiver of the target child was asked to answer a structured questionnaire through an interview with the surveyor. This questionnaire was divided into three sections; the first section incorporated epidemiologic and sociodemographic characteristics of the child and his parents (age, gender, residence, maternal age, number of children in the family, and parental education and occupation) [25, 31]. The second section inquired about the perinatal history of the studied children and their mothers including maternal diseases during pregnancy, history of difficult labor, history of premature labor, low birth weight, or any postnatal problem such as cyanosis, jaundice, convulsions, meningitis, or admission to Neonatal Intensive Care Unit (NICU) for more than two days. Lastly, a third section was a developmental screening of the child’s developmental skills corresponding to his chronological age using *The revised Denver Prescreening Developmental Questionnaire (R-PDQ)*. Answering the structured questionnaire lasted for 20 min. It was designed as multiple-choice questions filled by the surveyor according to the answers of the caregiver. The surveyors had received a high quality training on administration of this questionnaire prior to implementation of the survey.

*R-PDQ* has been revised to update the monitoring and screening of children’s development. It was developed by modifying the DDST items into questions that can be answered with “YES” or “NO” by the caregiver [33]. Being simpler than the DDST, the R-PDQ reduces the cost of developmental screening. It also involves parents and awakens their interest in their child’s development. Four sectors are assessed: Personal-social (PS), Fine motor-adaptive (FM), Language (LA), and Gross motor (GM). It is a cheap, quick, and practical first-step screen to be used in community mass screening programs. Questions are categorized according to age into 4 questionnaires for 0–9 months, 9–24-month, 2–4 years, and 4–6-year-old children. It uses the same norms as the DDST-II [34]. The answer to each question can be normal (which means the child is able to do the task), delayed (which means the child is not able to do the task that 90% of his/her age-matched children can do) and caution (which means the child is not able to do the task that 75% of his/her age-matched children can do [35, 36]. R-PDQ has good content validity and reliability and moderate sensitivity and specificity in comparison with the DDST-II [37]. In the current study, the Arabic-adapted version was used [38].

*Denver Developmental Screening Test, 2nd edition (DDST)* [34, 35] is used to screen children who are at risk of developmental delays from 1 month to 6 years of age, to confirm suspected problems with an objective measure. The test can be easily administered in about 20 min and scoring is based on the investigator’s observation and parental reporting in four areas of functioning: fine motor-adaptive, gross motor, personal-social, and language skills. The ease of use and simplicity of this screening tool make it advantageous around the world [39]. The test has good inter-rater and test–retest reliability (correlations 0.90 or higher for most tests) [40]. The test is valid and there is a strong relationship between classification on the DDST and scores on the Stanford-Binet intelligence scales and the earlier edition of the Bayley infant scales [41].

It is a standardized measure that has been normed on a diverse sample. The norms indicate when 25%, 50%, 75%, and 90% of children passed each item. Each item is scored as pass, fail, or refused. Items that can be completed by 75% of children but are failed by the examined child are called cautions; items that can be completed by 90% of children but are failed by the examined child are referred to as delays. If the rescreening results remained as suspect or untestable, referral for further evaluation was done.

### Survey implementation and flow of work

Before the implementation of phase one and two, and due to the limited numbers of psychologist in the majority of the selected areas, 208 selected nurses were trained by specialized consultants (26 nurses per each governorate) to ensure using the screening tests for development and calculate the score. At the same time, the surveyors from the Cairo Demographic Center (CDC) had received a high-quality training on administration of the structured questionnaire. A pilot study was performed on 80 participants before the implementation of the screening (10/governorate) to ensure the validity of the questionnaire items through revising and modifying difficulty- understood items and then re-introduced them.

In the current study, a two-step screening procedure was adopted for the assessment of developmental status of the targeted huge sample size (21,316 children). The range of the targeted houses per governorate was from 1960–4170 (S Table 1). The screening phases were done under the supervision of a collaborative team from the (CDC) with professional team members from the MOHP and NRC. The advantage of this two-step process is to reserve the DDST-II for children who are likely to receive suspect Denver-II scores.

The revised-Denver Prescreening Developmental Questionnaire (R-PDQ) [33] was used as a first-step screen on house-to-house basis. Each surveyor targeted an average of 6 houses per day for an average of 5 months. Only children who were suspect on the first screen, underwent screening on the longer DDST-II [34]. The advantage of this two-step process is to reserve the DDST-II for children who are likely to receive suspect Denver-II scores.

Children with suspected developmental delays on DDST were retested 2 weeks later to rule out temporary considerations as fatigue, fear, or illness. An overall mean agreement between parental responses on R-PDQ and the results of DDST was 93.3%. When the rescreening results on the DDST-II remained as suspect or untestable, children were referred to specialized team to confirm the diagnosis. A multidisciplinary team involved a wide variety of professionals from the Ministry of Health and Population (MOHP) and the National Research Centre (NRC), thoroughly assess the referred children with suspect diagnosis.

Developmental behavioral pediatricians were the leaders of the team which involved, clinical psychologists, Speech and Language therapists, occupational therapists and social-care professionals. They collaborated to confirm the diagnosis of motor, language, personal-social or global developmental delay.

Finally, the child was placed in one of two categories; either normal or delayed. With 2.5% losses and 10.5% negative diagnosis, children who ascertained the diagnosis of developmental delay have reached 87% of those who compiled referrals.

The implementation of both phase one and phase two was conducted over one year in a simultaneous way along the geographical areas of the eight randomly selected governorates.

### Statistical analysis

Data was entered into the Household Registration System (HRS) version 2.1. Data were analyzed using Statistical Package for the Social Sciences (SPSS) version 24.0 software (IBM SPSS Statistics for Windows, Version 24.0. Armonk, NY: IBM Corp.) [42]. All data were presented by percentages. The denominator for the calculation of the proportion of the studied variables was the total number of children enrolled in the study (*n* = 21,316). Missed values were not excluded from the denominator but they were excluded from the nominator during proportion calculation. Comparison between each of the DDs’ domains and children without delay was done using odds ratios (OR) and 95% confidence intervals (CI) as two steps. Factors that were found to be statistically significant in the univariate logistic regression analysis were subjected to multivariate logistic regression (Enter Wald) for adjusting and controlling the effect of confounding variables to determine the predictors for at least DD and domain‑dependent. Results were presented in terms of crude odds ratio (COR) and adjusted odds ratio (AOR) in a univariate and multivariate analysis respectively. Test–retest and interrater evaluation were used in the pilot phase, in order to determine the reliability of the used two tests by Kappa statistic.

A Multivariate Logistic regression analysis was done to predict the odds of developing any of the DDs based on the values of the independent variables (risk factors for each domain of DDs and at least one DD. A significant association is considered if the 95% CI does not include the value 1.0, and a cutoff *p*-value of less than 0.05 is used for all tests of statistical significance in this study.

## Results

Table 1 shows the characteristics of the study population who were 21,316 children aged 1—< 6 years. Children were nearly equally distributed between social classes, rural and urban localities. They were distributed among governorates proportional to population size. The surveyed boys were slightly higher than girls. Regarding the age distribution, children aged 3- < 6 years represented nearly 60% versus children aged 1- < 3 years who represented 39% of the whole sample. Most of the mothers were giving birth in the of 18—< 35 age range (86.3%). The highest percentage of mothers and fathers had high school education or technical and above intermediate (44.3% and 46.3% respectively). Most of the mothers were unemployed (86%). Houses headed with single mothers were 4.1% versus 0.5% headed with single fathers. Among the perinatal problems, the presence of neonatal jaundice was the most prevalent (29.0%) followed by difficult labor (16.1%).

**Table 1:** Characteristics of the study population

Characteristics	Surveyed children (21,316)	
	**Number**	**Column %**
Locality		
Urban	9707	45.5
Rural	11,609	54.5
Social class		
Low	6924	32.5
Middle	7093	33.3
High	7299	34.2
Geographical distribution		
Cities	3264	15.3
Lower Egypt	7921	37.2
Upper Egypt	7705	36.1
Frontier	2426	11.4
Sex		
Boys	11,076	52.0
Girls	10,240	48.0
Age categories of children		
1- < 3 years	8383	39.3
3- < 6 years	12,933	60.7
Mother’s age at giving birth		
< 18 years^a^	891	4.2
18 to < 35 years	18,382	86.3
> 35 years	1939	9.1
Mothers’ Education		
Illiterate/ Read & write/ Primary/ Prep	8150	38.2
High School & technical/ above intermediate	9432	44.3
University or higher	3622	17.0
Fathers’ Education		
Illiterate/ Read & write/ Primary/ Prep	7129	33.4
High School & technical / Above intermediate	9867	46.3
University or higher	3449	16.2
Mother’ s work		
Employed (paid-unpaid-his own-employer)	2976	14.0
Unemployed / does not look for work	18,225	86
Families with single parent		
Single mothers	870	4.1
Single fathers	109	0.5
Perinatal problems		
Children with at least one perinatal problem	7096	33.3
Premature delivery (< 37 weeks gestation)	229	1.1
Low birth weight (< 2500 mg)	1077	5.1
Children suffered from jaundice after birth	6180	29
Children suffered from cyanosis after birth	313	1.5
Children suffered from any convulsions	355	1.7
Children were admitted to an incubator for more than two days	1770	8.3
Children suffered from meningitis	190	0.9
Maternal problems		
Mothers had any health problems during pregnancy^b^	1500	7.0
History of difficult labor^c^	3435	16.1

^a^This category was set down because 17% of Egyptian girls are married before their 18th birthday [43]

^b^Mothers having complications during pregnancy such as gestational diabetes, hypertension, iron deficiency anemia, anxiety, depression, or infection [44]

^c^Difficult labor refers to prolongation in the duration of labor, especially in the first stage of labor. It can be a contributor to maternal mortality and morbidity if unrecognized or untreated [45]

The total number of the surveyed children was 21,316. The prevalence of the typically developing children were 93.6% versus children with at least one DDs which was 6.4% (1365 children), 1.9% of them with single delay (412 children), and 4.5% (953 children) with global DDs. Overall maximum DD was recorded in the Language domain (4.2%), 2.04% of children had a fine motor delay, while 1.9% had a gross motor delay, and (3.2%) had a personal social delay (Fig. 1).

Table 2 shows the odds of having DDs according to the sociodemographic characteristics. The odds for the presence of at least one delay were significantly higher among urban than that in rural communities (COR = 1.28, 95% CI: 1.14–1.42), and was nearly one and half times significantly higher in the middle social class than that of low and high social class (COR = 1.49, 95% CI: 1.30–1.70 & COR = 1.40, 95% CI: 1.23–1.59 respectively).

**Table 2:** Odds of having developmental delay among children aged 1- < 6 years according to the sociodemographic characteristics

**Socio-demographic parameters** ***N*** ** = 21,316**	**Gross Motor** **(GM)** **N (%)** **412 (1.9)**	**Fine Motor-adaptive (FMA)** **N (%)** **435 (2)**	**Language** **(L)** **N (%)** **904 (4.2)**	**Personal-Social (PS)** **N (%)** **681 (3.2)**	**Children with at least one delay** **N (%)** **1365 (6.4)**
**Locality**					
**Urban (** ***n*** ** = **9707)	209 (2.2)	225 (2.3)	476 (4.9)	349 (3.6)	699 (7.2)
**Rural (** ***n*** ** = **11,609)	203 (1.7)	210 (1.8)	428 (3.7)	332 (2.9)	666 (5.7)
**Odds (CI)** ** OR urban vs. rural**	**1.24** (1.02–1.50)*	**1.29** (1.07–1.56)*	**1.35** (1.18–1.54)*	**1.27** (1.09–1.48)**	**1.28** (1.14–1.42)**
**Social class**					
**Low (** ***n*** ** = **6924)	118 (1.7)	115 (1.7)	314 (4.3)	187 (2.7)	379 (5.5)
**Middle (** ***n*** ** = **7093)	170 (2.4)	176 (2.5)	347 (4.9)	298 (4.2)	562 (7.9)
**High (** ***n*** ** = **7299)	124 (1.7)	144 (2)	314 (4.3)	196 (2.7)	424 (5.8)
**OR middle vs. low**	**1.42** (1.12–1.80)*	**1.51** (1.19–1.91)*	**1.41** (1.196–1.67)*	**1.58** (1.31–1.90)**	**1.49** (1.30–1.70)**
**OR middle vs. high**	**1.421** (1.13–1.80)*	**1.26** (1.01–1.58)*	1.14 (0.98–1.34)	**1.59** (1.32–1.91)**	**1.40** (1.23–1.59)**
**OR low vs. high**	1.003 0.78–1.29	.839 0.66–1.08	.81 0.68–0.96	1.01 0.82–1.23	0.94 0.81–1.08
**Geographical distribution**					
**Cities (** ***n*** ** = **3264)	91 (2.8)	79 (2.4)	216 (6.6)	149 (4.6)	313 (9.6)
**Lower Egypt (** ***n*** ** = **7921)	112 (1.4)	160 (2)	324 (4.1)	271 (3.4)	520 (6.6)
**Upper Egypt (** ***n*** ** = **7705)	168 (2.2)	149 (1.9)	282 (3.7)	210 (2.7)	418 (5.4)
**Frontier (** ***n*** ** = **2426)	41 (1.7)	47 (1.9)	82 (3.4)	51 (2.1)	114 (4.7)
**OR lower Egypt vs. cities**	**0.50** (0.38–0.66)**	0.83 (0.63–1.09)	.60 (0.50–0.72)	**0.74** (0.60–0.91)**	**0.66** (0.57–0.77)**
**OR upper Egypt vs. cities**	0.78 (0.60–1.01)	0.80 (0.60–1.05)	0.54 (0.45–0.64)	**0.59** (0.47-.0.73)**	**0.54** (0.46–0.63)**
**OR frontiers vs. cities**	0.599 (0.41–0.87)	0.80 (0.55–1.15)	0.49 (0.38–0.64)	**0.45** (0.33–0.62)**	**0.47** (0.37–0.58)**
**OR lower Egypt vs. frontiers**	0.83 (0.58–1.97)	1.04 (0.75–1.45)	1.22 (0.95–1.56)	**1.65** (1.22–2.23)**	**1.43** (1.16–1.75)**
**OR upper Egypt vs. frontiers**	1.297 (0.92–1.83)	0.998 (0.72–1.39)	1.09 (0.85–1.395)	1.30 (0.96–1.78)	1.16 (0.94–1.44)

^*^ *p*-value significant at < 0.05

^**^ *p*-value highly sig at < 0.01

Regarding the different types of developmental delay, living in urban communities carried significantly higher odds for delay in all domains. Belonging to middle class carried significantly one and a half higher odds for all types of delay than low social class or high social class except for language domain in high class.

Living in geographical areas other than cities appeared to decrease the risk for DD. Living in lower Egypt significantly decreased the odds of gross motor and personal-social delay by 50% and 25%, respectively, than in cities. (COR = 0.50, 95% CI: 0.38–0.66 for gross motor; COR = 0.74, 95% CI: 0.60–0.91 for personal-social). Meanwhile, living in upper Egypt and the frontier was significantly associated with decreased odds for personal-social delay by 40% and 55% respectively than living in cities (COR = 0.59, CI: 0.47–0.73 & COR = 0.45, 95% CI: 0.33–0.62 respectively).

Concerning the epidemiological factors (Table 3), children aged 3– < 6 years were the most likely to be significantly diagnosed with any DD than children aged 1- < 3 years, with the highest odds for personal-social (COR = 3.55, 95% CI: 2.89–4.37) followed by Fine Motor delay (COR = 3.45, 95% CI: 2.67–4.46). Boys were nearly one and a half more likely than girls to be diagnosed with any DD for all domains except for Gross motor delay (COR = 1.18, 95% CI: 0.97–1.44).

**Table 3:** Odds of having developmental delay among children aged 1- < 6 years according to epidemiological characteristics

**Epidemiological characteristics** ***n*** ** = 21,316**	**Gross Motor** **(GM)** **N (%)** **412 (1.9)**	**Fine Motor-adaptive (FMA)** **N (%)** **435 (2)**	**Language** **(L)** **N (%)** **904 (4.2)**	**Personal-Social (PS)** **N (%)** **681 (3.2)**	**Children with at least one delay** **N (%)** **1365 (6.4)**
**Age categories of Children (years)**					
1- < 3 years (*n* = 8383)	131 (1.6)	70 (0.84)	265 (3.2)	108 (1.3)	349 (4.2)
3- < 6 years (*n* = 12,933)	281 (2.2)	365 (2.8)	639 (4.9)	573 (4.4)	1016 (7.9)
COR Odds (CI) 3- < 6 years vs. 1- < 3 years	1.40 (1.13–1.72)*	3.45 (2.67–4.46)**	1.59 (1.38–1.84)*	3.55 (2.89–4.37)**	1.96 (1.73–2.22)**
**Sex**					
Male (*n* = 11,076)	231 (2.1)	266 (2.4)	595 (5.4)	410 (3.7)	855 (7.7)
Female (*n* = 10,240)	181 (1.8)	169 (1.7)	309 (3)	271 (2.6)	510 (5)
COR males/Females CI	1.18 (0.97–1.44)	1.47 (1.21–1.78)*	1.83 (1.59–2.10) *	1.41 (1.21–1.65)**	1.60 (1.43–1.79)**
**Mother’s age at giving birth (years)**					
< 18 years (n = 891)	19 (2.1)	14 (1.6)	44 (4.9)	35 (3.9)	74 (8.3)
18 to < 35 years (n = 18,382)	341 (1.9)	361 (2)	767 (4.2)	569 (3.1)	1156 (6.3)
> 35 years (n = 1939)	45 (2.3)	54 (2.8)	83 (4.3)	71 (3.7)	124 (6.4)
COR ≥ 35 vs. < 18 CI	1.09 (0.63–1.88)	1.80 (0.10–3.25)	0.86 (0.59–1.25)	0.93 (0.62–1.40)	0.75 (0.56–1.02)
COR 18- < 35 vs. < 18 CI	0.87 (0.54–1.38)	1.26 (0.73–2.15)	0.84 (0.61–1.15)	0.78 (0.55–1.11)	0.75 (0.58–0.95)*
COR 18- < 35 vs. ≥ 35 CI	0.80 (0.58–1.09)	0.70 (0.52–0.93)*	0.97 (0.77–1.23)	0.84 (0.65–1.08)	0.98 (0.81–1.19)
No mother (*n* = 109)	7 (6.4)	6 (5.5)	10 (9.2)	7 (6.4)	12 (11)
COR single father vs. both parents at home	3.91 (1.80–8.48)*	3.13 (1.36–7.18)**	2.55 (1.32–4.93)**	2.09 (0.97–4.51)	1.82 (0.99–3.32)
No father (*n* = 870)	16 (1.8)	24 (2.8)	50 (5.7)	34 (3.9)	62 (7.1)
COR single mother vs. both parents at home (CI)	0.95 (0.57–1.57)	1.38 (0.91–2.099)	1.34 (1.04–1.88) *	1.24 (0.88–1.77)	1.13 (0.87–1.47)

^*^ *p*-value significant at < 0.05

^**^ *p*-value highly sig at < 0.01

The age of mothers at giving birth significantly influenced the fine motor domain. Mothers giving birth at the age range 18 to < 35 years were significantly associated with decreased odds of having children with fine motor delays by 30% (COR = 0.70, 95% CI: 0.52–0.93) than mothers giving birth at age > 35 years. Single-parent families implicated the risk of DD, living with single fathers carried increased odds of having a gross motor, fine motor, and language delay by nearly three times, meanwhile, living with single mothers carried odds for Language delay only.

The odds of having DD among children aged 1- < 6 years were the least with higher maternal and paternal education (Table 4). Children with mothers or fathers who had higher education were less likely to have any type of delays especially when parents had college or greater education level as it was noted that these children had less odds (from 27 to 49%) for varied domains compared to lower grades of education. The influence of paternal education was nearly equal to that of maternal education. Mothers’ work does not affect the odds of having any delay.

**Table 4:** Odds of having developmental delay among children aged 1- < 6 years according to parental characteristics

**Socio-demographic parameters** ***n*** ** = 21,316**	**Gross Motor** **(GM)** **N (%)** **412 (1.9)**	**Fine Motor -adaptive (FMA)** **N (%)** **435 (2)**	**Language** **(L)** **N (%)** **904 (4.2)**	**Personal-Social (PS)** **N (%)** **681 (3.2)**	**Children with at least one delay** **N (%)** **1365 (6.4)**
**Mother’s Education**					
1. Illiterate/ below high school (*n* = 8150)	204 (2.5)	200 (2.5)	380 (4.7)	291 (3.6)	579 (7.1)
2.High School (*n* = 9432)	151 (1.6)	183 (1.9)	391 (4.1)	294 (3.1)	593 (6.3)
3.University or higher (*n* = 3622)	50 (1.4)	46 (1.3)	122 (3.4)	89 (2.5)	181 (5)
COR 2 vs. 3 CI	1.54 (1.11–1.97) *	1.23 (0.97–1.63)	1.19 (0.81–1.74)	1.28 (1.00–1.62) *	1.28 (1.08–1.51) **
COR 2 vs. 1 CI	0.79 (0.64–0.96) *	.92 (0.77–1.10)	0.69 (0.54–0.89) *	0.87 (0.74–1.02)	0.88 (0.78–0.99) *
COR 3 vs. 1 CI	0.51 (0.37–0.71) **	0.73 (0.56–0.95) *	0.58 (0.40–0.85) **	0.68 (0.53–0.87) **	0.69 (0.58–0.82) **
**Father’s Education**					
1.Illiterate/ below high school (*n* = 7129)	192 (2.7)	195 (2.7)	361 (5.1)	273 (3.8)	538 (7.6)
2.High School (*n* = 9867)	165 (1.7)	174 (1.8)	394 (4)	301 (3.1)	613 (6.2)
3.University or higher (*n* = 3449)	39 (1.1)	42 (1.2)	99 (2.9)	73 (2.1)	152 (4.4)
COR 2 vs. 3 CI	1.49 (1.05–2.11) *	1.46 (1.04–2.04) *	1.41 (1.13–1.76) *	1.46 (1.12–1.88) **	1.44 (1.20–1.72) **
COR 2 vs. 1 CI	0.61 (0.50–0.76) *	0.64 (0.52–0.79) *	0.78 (0.67–0.90) *	0.79 (0.67–0.93) **	0.81 (0.72–0.92) **
COR 3 vs. 1 CI	0.41 (0.29–0.58) **	0.44 (0.31–0.61)**	0.55 (0.44–0.70) *	0.54 (0.42–0.71) **	0.57 (0.47–0.68) **
**Mother’ s work**					
1. Employed (*n* = 2976)	47 (1.6)	58 (1.9)	134 (4.5)	87 (2.9)	186 (6.3)
2. unemployed (*n* = 18,225)	357 (2)	371 (2)	759 (4.2)	587 (3.2)	1167 (6.4)
COR 2 vs. 1 CI	1.25 (0.92–1.69)	1.05 (0.79–1.38)	.922 (0.76–1.11)	.48 (0.22–1.05)	1.03 (0.88–1.20)

^*^ *p*-value significant at < 0.05

^**^ *p*-value highly sig at < 0.01

The Odds of having developmental delay for all the studied domains were significant for all the studied medical perinatal history problems as shown in Table 5. The odds for DDs were almost three times for low-birth-weight babies (baby’s weight less than 2.5 kg at birth) than children with normal birth weight, with the highest odds for GM (COR = 3.90, 95% CI: 2.98–5.09) and FM (COR = 3.52, 95% CI: 2.69–4.60) delays. Whereas children of mothers having any health problem during pregnancy carried more than twice the odds for DDs than children born to healthy mothers. Difficult labor carried one and a half odds for all types of DDs. All the studied postnatal child problems (postnatal cyanosis, postnatal convulsions, postnatal meningitis, etc.) carried higher odds for all the studied DDs domains with a range of 6 to 8 times more odds for children suffered from convulsions (COR = 8.15, 95% CI: 5.86–11.3 for GM, COR = 7.23, 95% CI: 5.18–10.1 for FM & COR = 6.10, 95% CI: 4.53–8.20 for PS) or cyanosis after birth (COR = 6.89, 95% CI: 4.78–9.93 for GM & COR = 5.61, 95% CI: 3.82–8.23 for FM). Children who suffered from meningitis after birth carried the highest odds for FM (COR = 5.87, 95% CI: 3.66–9.43). A child who was kept in NICU for more than two days carried the highest odds for PS (COR = 2.79, 95% CI: 2.29–3.39) followed by FM (COR = 2.60, 95% CI: 2.03–3.33).

**Table 5:** Odds of having developmental delay according to medical perinatal history and postnatal child problems

Type of the risk^a^	Total children Surveyed *N* = 21,316			
	**Gross Motor** **(GM)** **N (%)** **412 (1.9)**	**Fine Motor-adaptive (FMA)** **N (%)** **435 (2)**	**Language** **(L)** **N (%)** **904 (4.2)**	**Personal-Social (PS)** **N (%)** **681 (3.2)**
Mother had any health problem during pregnancy (***n ***= 1500)	69 (4.6)	74 (4.9)	131(8.7)	113 (7.5)
** COR** ** (CI)**	2.74 (2.10–3.57)**	2.80 (2.17–3.61)**	2.36 (1.94–2.86)**	2.76 (2.24–3.40)**
Difficult labor (***n*** = 3435)	90 (2.6)	101 (2.9)	182 (5.3)	153 (4.5)
** COR** ** (CI)**	1.47 (1.16–1.86)*	1.59 (1.27–1.99)	1.33 (1.13–1.57)*	1.53 (1.27–2.84)**
Child born less than 37 weeks (preterm pregnancy) (***n*** = 229)	10 (4.4)	14 (6.1)	25 (10.9)	19 (8.3)
** COR** ** (CI)**	2.35 (1.24–4.46)**	3.20 (1.85–5.54)**	2.82 (1.85–4.29)**	2.79 (1.74–4.50**
Baby’s weight less than 2.5 kg at birth (***n*** = 1077)	68 (6.3)	66 (6.1)	107 (9.9)	89 (8.3)
** COR** ** (CI)**	3.90 (2.98–5.09)**	3.52 (2.69–4.60)**	2.69 (2.18–3.33)**	2.99 (2.37–3.77)**
Child suffered from jaundice after birth (***n*** = 6180)	145 (2.3)	158 (2.6)	339 (5.5)	270 (4.4)
** COR** ** (CI)**	1.34 (1.09–1.64)*	1.41 (1.16–1.72)*	1.50 (1.30–1.72)*	1.63 (1.40–1.91)**
Child suffered from cyanosis after birth (***n*** = 313)	35 (11.2)	31 (9.9)	50 (16.0)	38 (12.1)
** COR** ** (CI)**	6.89 (4.78–9.93)**	5.61 (3.82–8.23)**	4.49 (3.29–6.12)**	4.38 (3.09–6.21)**
Child suffered from any convulsions (***n*** = 355)	45 (12.7)	43 (12.1)	67 (18.9)	56 (15.8)
** COR** ** (CI)**	8.15 (5.86–11.3)**	7.23 (5.18–10.1)**	5.59 (4.25–7.36)**	6.10 (4.53–8.20)**
Child was admitted to an incubator for more than two days (***n*** = 1770)	69 (3.9)	81 (4.6)	152 (8.6)	132 (7.5)
** COR** ** (CI)**	2.27 (1.75–2.96)**	2.60 (2.03–3.33)**	2.35 (1.96–2.82)**	2.79 (2.29–3.39)**
Child suffered from meningitis (***n ***= 190)	14 (7.4)	20 (10.5)	26 (13.7)	21 (11.1)
** COR** ** (CI)**	4.14 (2.38–7.20)**	5.87 (3.66–9.43)**	3.66 (2.40–5.56)**	3.85 (2.43–6.10)**

^a^row %

^*^ *p*-value significant at < 0.05

^**^ *p*-value highly sig at < 0.01

Table 6 shows the data of the multivariate logistic regression model for exploring the predictors of DDs in different domains among preschool children aged 1- < 6 years. Seventeen significant variables that were associated with the presence of at least one DD versus healthy children (as evident from the univariate analysis) were entered into a multivariate logistic regression model using the enter selection procedure to explore the predictors of each of the studied DD domains. These variables included: eight sociodemographic/epidemiological, two maternal, and seven children risk factors.

**Table 6:** Multivariate Logistic regression model for prediction of DD vs. children without delay: gross motor delay (GM), fine motor-adaptive delay (FMA), Language delay (L), Personal-social delay (PS), and Children with at least one delay

**Parameters**	**Gross Motor** **(GM)**		**Fine Motor-adaptive (FMA)**		**Language** **(L)**		**Personal-Social (PS)**		**Children with at least one delay**	
	**AOR**	**CI**	**AOR**	**CI**	**AOR**	**CI**	**AOR**	**CI**	**AOR**	**CI**
**Sex (male is the base)**	1.22	0.9–1.50	1.47	1.20–1.81*	1.84	1.59–2.14*	1.37	1.17–1.62**	1.60	1.42–1.80**
**Locality (urban is the base)**	1.14	0.9–1.47	1.30	1.02–1.65*	1.14	0.95 – 1.35	1.24	1.02–1.50*	1.13	.98–1.30
**Social level (middle is the base)**	1.35	1.08–1.67*	1.30	1.02–1.65*	1.24	1.06–1.45*	1.59	1.35–1.87**	1.48	1.28 -1.71**
**Geographical distribution: (Lower, Upper, and Frontiers) are the base**										
Lower	0.64	0.50–0.91*	1.199	0.85–1.69	0.65	0.52–0.82*	0.80	0.66 -0.97*	0.82	0.71–0.94**
Upper	1.0	0.70–1.35	1.117	0.79–1.59	0.59	0.47–0.74*	0.66	0.47 -0.91*	0.75	0.60–0.94*
Frontiers	0.69	0.453–1.03	1.058	0.71–1.60	0.54	0.41–0.72*	1.07	0.83 -1.38	1.38	1.15–1.67**
**Maternal education: University and above is the base**										
University and above	0.59	0.47–0.74*	0.60	0.40–0.74*	0.82	0.70–0.96*	0.93	0.70–1.23	0.83	0.68–1.03
**Paternal education: Less education is the base**										
Less educated	1.83	1.21–2.77*	1.45	0.98–2.16	1.57	1.20–2.05	1.60	1.19–2.17**	1.55	1.25–1.92**
**Maternal problems are the base**										
Mothers had any health problems during pregnancy	1.95	1.43–2. 67**	1.91	1.40–2.60*	1.74	1.39–2.19**	1.99	1.57–2.51**	1.73	1.44–2.07**
**Perinatal problems are the base**										
Baby’s weight is less than 2.5 kg at birth	2.63	1.91–3.63**	2.45	1.77–3.39**	1.74	1.35–2.24**	1.62	1.23–2.14**	2.06	1.69–2.52**
Children suffered from cyanosis after birth	2.94	1.83–4.72**	1.94	1.16–3.27**	1.99	1.33–2.95**	1.39	.88–2.19	1.77	1.26–2.49**
Children suffered from any convulsions	3.76	2.40–5.89**	2.56	1.58–4.15**	2.34	1.62–3.40**	2.79	1.88–4.12**	2.68	1.97–3.64**
Child was admitted to an incubator for more than two days	1.28	.92–1.79	1.54	1.13–2.10*	1.45	1.16–1.81*	1.77	1.40–2.23**	1.37	1.14–1.64**
Constant	0.007		0.001		0.021		0.012		0.001	

^*^ *p*-value significant at < 0.05

^**^ *p*-value highly sig at < 0.01

The final model had a good fit for seven variables increasing the association for at least one DD (three sociodemographic/epidemiological, two maternal, and two children risk factors). The strong predictors for at least one DDs and for all DDs were in order: children suffering from convulsions (AOR = 2.68; 95% CI: 1.97–3.64), born with LBW (AOR = 2.06; 95% CI: 1.69–2.52), or if mothers having any health problem during pregnancy (AOR = 1.73; 95% CI: 1.44–2.07). Belonging to the middle social class was also a predictor for all the domains of developmental delays carrying almost one a and half odds for all delays. Children kept in an incubator for more than two days was a predictor for all domains except GM. Whereas higher maternal education decreases the odds to have any delay by a range of 41% for the gross motor domain (AOR = 0.59, 95% CI:0.47–0.74) and 18% for Language (AOR = 0.82, 95% CI:0.70–0.96). Low paternal education was accompanied by nearly twice the odds for GM (AOR = 1.83, 95% CI:1.21–2.77), PS (AOR = 1.60, 95% CI:1.19–2.17), and nearly one and a half odds for at least one delay (AOR = 1.55, 95%CI:1.25–1.92). Living in Frontiers decreases the odds to have Language delay by nearly 50% (AOR = 0.54, CI: 0.41–0.74) than living in cities. Living in lower Egypt decrease the odds to have both GM and L by nearly 35% (AOR = 0.64, 95% CI:0.50–0.91 & AOR = 0.65, 95% CI:0.52–0.82 respectively) than living in cities. Whereas living in upper Egypt decreases the odds to have only L delay by nearly 40% (AOR = 0.59, 95% CI:0.47–0.74) than living in cities. Living in urban communities carried significantly high odds for FM and PS delay than living in rural communities (AOR = 1.30, 95% CI:1.02–1.65, AOR = 1.24, 95% CI: 1.02–1.50 respectively).

## Discussion

Detecting developmental delays and their determinants early in life will help in the provision of minimum interventions that are needed for reversal and sustainable gains allowing for healthy life and wellbeing.

The current study revealed a 6.4% prevalence of DD among children aged 1- < 6 years. National health surveys of different countries reported wide variability in the prevalence of DDs ranging from 10% in Europe and Central Asia to 42% in West and Central Africa. This was attributed to enormous variations in skills of the literacy-numeracy domain that was examined among 3 other domains in the study involved 63 low- and middle-income countries [46]. When comparing the prevalence of DD, it was found that age, study type, region, and operational definition of DD versus developmental disability, are important factors affecting prevalence estimates. WHO reported a prevalence of preschool children’s developmental delay in low- and middle-income countries by 17% in Senegal, 15% in Nigeria, 13% in India, and 24% in Brazil [47]. A study in one of the Arab countries (Saudi Arabia), has reported an overall prevalence of 16.4% [48]. This relatively high prevalence in Saudi children was explained by the early introduction of complementary food before 6 months of age, narrow spacing between children, and low maternal education among the studied sample. In the current study, authors examined a representative sample of preschool children, used screening tools of moderate sensitivity and specificity and highly efficient confirmatory stage, and the research was confined to children with a developmental delay not disabilities, so the reported prevalence seems less than that of other developing countries. In Egypt, no previous National studies assessing the prevalence of developmental delay were recorded. Previous Egyptian studies conducted their research within specific governorates or localities, or studied specific groups, others studied predictors of delay in specific domains among young infants as cognitive, language, or socio-emotional domain [2–4, 22, 49].

Concerning the prevalence of the studied domains in the present study, the highest prevalence of DD was in the Language domain (4.2%) and the least was in the gross motor domain (1.9%). Again, the current prevalence of the two domains was lower than that reported by other Egyptian and non-Egyptian studies. The prevalence of Language Delay in a previous Egyptian study (was 34.4%) which is very high, because the population in this study was children having intellectual disability [50]. A worthy note to mention is that the present study was a representative study enrolling children from different social classes, rural and urban localities distributed among governorates proportional to population size making the results more accurate.

In the present study, the prevalence of delay in personal-social was 3.2%. This finding agrees with the prevalence of delay in personal – social and communication domains estimated in apparently normal preschool children at the entry to kindergarten in Isfahan, it was 2.2% and 1.2% respectively [49]. On the contrary, *Sharma and his colleagues in 2019* reported a delay in personal-social domains by 8.9% [20]. They explained this high prevalence by the low parental education and income in rural areas where the study was conducted.

The delay in the fine motor and the gross motor domains in the current study were 2% and 1.9% respectively. This accords with a cross-sectional Egyptian study that found no Gross motor delay versus 1.7% fine motor delay among the studied preschool children [22].

This study revealed that 4.5% of children aged 1- < 6 years were suffering from global DD (GDD). The exact prevalence of GDD is not precisely estimated; different studies estimated prevalence in a range from 1–3% [51, 52]. However, one Saudi Arabia study reported 51% GDD in the studied children and this was explained by the high rate of consanguinity and small sample size [53]. It was noted that fine motor delay and personal-social delay were commonly to be combined with other delays and rarely to be detected as a single delay. So, when either delay is detected, the examiner has to search for underlying delays in other domains. Correspondingly, the personal-social delay may be due to delayed language development [54, 55].

Regarding the influence of socio-demographic distribution on the prevalence of DDs domains; it has been found that living in frontiers, Upper or Lower Egypt was a protective factor against any type of DDs than living in cities, (OR = 0.47, 95% CI: 0.37–0.58, OR = 0.54, 95% CI: 0.46–0.63, OR = 0.66, 95% CI: 0.57–0.77 respectively). This protective effect was highly significant against personal-social delay. In cities; exposure of young children to environmental pollution, living in narrow places with overcrowding, and bad hygienic conditions could be the causes of serious affection on physical development, impact on brain structures and functions, and in turn lead to DD [56, 57]. These reasons also explain why the prevalence of DDs in the current study was higher among urban than rural communities as proved by the multivariate logistic regression model. On the contrary, other studies reported higher risk for DDs among children living in rural areas [19, 23, 51, ].

Belonging to the middle social class was a predictor for delay in all developmental domains and was associated with almost one and a half odds for all delays than belonging to low or high classes. On the contrary, results of previous studies are constantly in favor of the more privileged children in all areas of development [58, 59]. Comparable to our finding, a recent cross-sectional Egyptian study reported that one-fourth of the children of the middle social class had below-average scores in all developmental domains on the Bayley III scales, which was explained by defective parenting [60]. This may be due to the current situation in Egypt. Throughout the last decade, Egyptians faced successive economic challenges, but the middle class suffers the most [61] Individuals of the middle class as well as those of the low class are confronted with struggles in basic services, such as employment, housing, food security, healthcare and education. However, middle class members do not benefit of the social protection programs initiated by the government. The current governmental policies focus on enhancing the living conditions of the poor and the most deprived classes in the slums [62], while the middle class is still out of the picture. Parents in this class are usually under pressure which affects parent–child relationship, middle class women, are trying hard to maintain the status of their households and not fall into poverty [63]. In addition, the rising inflation rates are likely to lead to reduction in food consumption patterns which may lead to malnutrition and affecting physical and cognitive development [64].

Concerning the influence of the epidemiological characteristics on the prevalence of DDs, in this study boys were nearly one and a half more likely than girls to be diagnosed with any delay for all domains except for Gross motor delay (OR = 1.18, CI: 0.9–1.44). The vulnerability of boys to DD is supposed to be due to the slow maturation of the nervous system among the male sex in addition to the perinatal testosterone effect on proper cell connection and cell death [65]. Boys and girls in the current study didn’t show a significant difference in gross motor skills. This finding is in accordance with previous studies [66, 67]. The presence of differences in fundamental motor skills between sexes could be attributed to a multidimensional interaction of environmental, socio-cultural, and genetic factors [68]. Other studies didn’t find a relation between sex and DD in different domains [69, 70].

Regarding the age distribution, in the current study children aged 3- 6 years who represented nearly two-thirds of the study sample, were the most likely to be significantly diagnosed with any DD than the younger group (1–3 years). Children aged 3–6 years are subjected to many risk factors such as; familial violence, lack of stimulating environment [71], chronic malnutrition with decreased immunity [72], and increased vulnerability to diseases [73, 74] due to defective weaning procedures or the unhealthy dietary behavior. Another explanation could be, developmental delay being more noticeable and overt in this age group as compared to younger children**.**

In the present study, maternal age at giving birth ranged from 18 to < 35 years significantly decreasing the odds of fine motor delay by 30% (OR = 0.70, 95%CI: 0.52–0.93) than mothers giving birth at age > 35 years. This may be due to the greater risk of chromosomal and congenital abnormalities as well as perinatal complications among pregnant women elder than 35 years [75]. A very important finding was that children living with single mothers/fathers were more subjected to DD. Living with single fathers was associated with odds of DD three times higher than children living with their mothers. Living with single mothers was associated with one and a half odds of DDs. Whereas all domains are affected by the mother’s absence, the father’s absence affected only the Language domain. It is well documented that home environment with the role of both parents as well as better parent–child interactions is positively correlated with children’s development [76].

Worldwide, many studies associated low parental education with developmental delay [77, 78]. In the present study, paternal education had a higher influence than maternal education. Children of parents with college or greater education levels were less likely to have any type of delay. Low education level is accompanied by decreased parental cognitive stimulation and responsiveness to cultural values and practices and acts as a barrier against receiving health education, and stimulatory learning activity sessions provided for improving children’s mental development [77]. Moreover, low levels of parental scholarly limited access to the internet and other social media. A recent study that was done in Egypt for assessing the effectiveness of different communication channels in rural communities found that the spread of social media was limited to only one-third of wives and their husbands [79]. At the same time, the present study found that Mothers’ work does not affect the odds of having any delay, data from other studies mentioned that early maternal employment initiates care by others as grandparents or baby sister or nursery which had a consequential effect on cognitive and behavioral development [80].

Despite the causes of DDs remaining unknown, perinatal problems were reported by this study as risk factors for DD in preschool children. Yet not all of these factors were proved as predictors of DDs as revealed by the multivariate logistic regression model. In the present study, children of mothers experiencing a health problem during pregnancy carried nearly twice the odds for DDs than children born to healthy mothers. As any maternal health problem can be complicated by fetal prematurity, low birth weight, distress, and asphyxia which are risk factors for developmental delay.

In the present study, all studied postnatal newborn problems carried higher odds for the studied DDs domains. Infants who suffered from convulsions had nearly three times the odds for DD in GM (OR = 3.76 95%CI: 2.40–5.89) and FM (OR = 2.56 95%CI: 1.58–4.15) domains. In agreement with Pappas and his colleagues 2016 [81] who found that moderate to severe neonatal encephalopathy contributes to cognitive impairment and developmental delay. The odds for DDs were almost more than two times for Low-birth-weight babies (LBW)- with the highest odds for GM (OR = 2.63 95%CI: 1.91–3.63) and FM (OR = 2.45 95% CI: 1.77–3.39). Higher risk of different types of DDs was also recorded in LBW compared to average weight [82, 83]. This may be due to common complications such as cerebral white matter injury, leukomalacia, and periventricular and\or intraventricular hemorrhage that are common among LBW [83]. In the current study, more than two days stay in the Neonatal Intensive care unit (NICU) was a predictor for delay in all domains except GM. It carried nearly twice the odds of delay for PS (OR = 1.77, 95%CI:1.40–2.23). This delay may be due to the absence of both maternal bonding and breastfeeding and the routine use of antibiotics during the newborn stay at NICUs. All these have a great impact on gut microbiota which in turn may be reflected in neurodevelopmental delay later [84]. When prioritizing the perinatal predictors for any delay in the current study, postnatal convulsion followed by LBW then cyanosis, maternal health problems during pregnancy and the least were children kept in an incubator for more than two days. The first three predictors share in causing either neurological defect or circulatory defect that will influence these sensitive growing brain cells which contribute to a delay in any of the domains.

Fortunately, most of the detected predictors are preventable and could be approached by holistic combined interventions that were proven to be effective. Proper antenatal care was documented by many Egyptian studies to positively exert its impact on birth outcomes [85, 86]. Raising mothers’ awareness about their rights for having perinatal care improved their utilization of the health care facilities with a positive effect on their health [87, 88]. On the other hand, investment in specific change agents like raising women’s awareness about environmental and hygienic issues was considered as a fundamental action for controlling environmental pollution and other risky hazards [89, 90]. Moreover, community-based parent-focused and child-focused interventions related to nutritional education proved to have a sustainable effect on child wellbeing in Egypt [91].

## Strengths

This study has several strengths. First: this study was done as a representative to all Egypt governorates, so the results could be generalized to the whole country. Second: the study accuracy is very high being conducted on a large sample size with a very small margin of error and high confidence level. Moreover, by design, the study considered the sociodemographic and economic information, so limited the expected confounding factors. Third: This study was the first study done in Egypt at a national level using multiple efficient procedures. Fourth: The study assessed child development at an early age starting from 1 year. Fifth: Our findings highlight both the key determinants and protective factors of the socioeconomic, epidemiological, and perinatal characteristics for each of the studied domains. Sixth: There was no possibility of recall bias, as the questions of both tests (R-PDQ and DDST-II) are concerned with the skills that the target child can do now, not in the past. If the child can do this skill the answer is yes or pass, if he can’t do it, the answer is No, or failed or no opportunity.

## Limitations

As this research is based on a cross-sectional population screening test, the study only focused on the risk factors that carry associations with each domain being delayed. Furthermore, causes resulting in developmental delay could not be investigated like other cross-sectional studies. On the other hand, the role of the environmental and genetic factors as contributing factors to developmental delays was not investigated and thus were not taken into account in the analysis. The relatively moderate sensitivity and specificity of the first screening test is thought to affect the efficiency of the study, however, the final outcome of DD prevalence and types relied on the second screening step and the confirmed diagnosis by the highly specialized team. Another limitation of this study was that Children normal on R-PDQ were not evaluated for any false negatives.

## Conclusion

This is the first national screening study estimating the prevalence of DD among preschool children, 6.4% of the investigated children had at least one DD, while 4.5% had two or more delays (GDD). The most prevalent delay was language delay. Perinatal factors are the most common determinants of DD in preschool children and the majority could be preventable risk factors followed by sociodemographic factors such as male gender, belonging to the middle social class, or living in cities/urban communities. High paternal education represents the most significant protective factor against developmental delay. Some of the detected predictors are preventable through perinatal care and health education.

### Supplementary Information

**Additional file 1: S Fig. 1.** Map of the 27 Egypt's governorates distributed within the four geographic regions (adapted using data from the Humanitarian Data Exchange under the CC BY-IGO license [31].

**Additional file 2: Supplementary Table 1.** List of the targeted Households (HH) according to the governorates, locality and sociodemographic status for screening of DD among children aged 1-<6 years.

**Additional file 3: S Table 2.** All data delay 1-6 years.

## Data Availability

All data generated or analyzed during this study are included in this published article (and its supplementary information files).
