# Supplementary material for: Screening and determinant of suspected developmental delays among Egyptian preschool-aged children: a cross-sectional national community-based study
Source: BMC Pediatr. 2023 Oct 19;23:521. doi: 10.1186/s12887-023-04335-0 (PMC10585886; doi:10.1186/s12887-023-04335-0)
Supplement: Supplementary file 1 — Additional file 1: S Fig. 1. Map of the 27 Egypt's governorates distributed within the four geographic regions (adapted using data from the Humanitarian Data Exchange under the CC BY-IGO license [31]. [file 12887_2023_4335_MOESM1_ESM.doc]

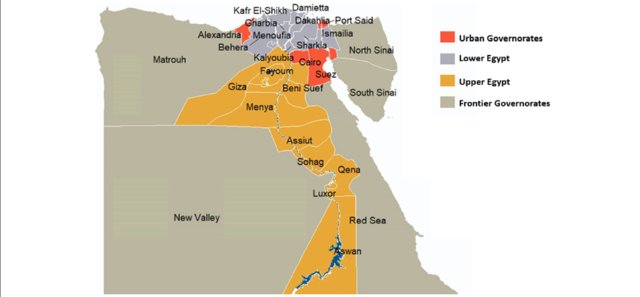


S Fig -1: Map of the 27 Egypt's governorates distributed within the four geographic regions (adapted using data from the Humanitarian Data Exchange under the CC BY-IGO license **[30]**
