# Supplementary material for: Screening and determinant of suspected developmental delays among Egyptian preschool-aged children: a cross-sectional national community-based study
Source: BMC Pediatr. 2023 Oct 19;23:521. doi: 10.1186/s12887-023-04335-0 (PMC10585886; doi:10.1186/s12887-023-04335-0)
Supplement: Supplementary file 2 — Additional file 2: Supplementary Table 1. List of the targeted Households (HH) according to the governorates, locality and sociodemographic status for screening of DD among children aged 1-<6 years. [file 12887_2023_4335_MOESM2_ESM.doc]

**Supplementary Table-1: List of the targeted Households (HH) according to the governorates, locality and sociodemographic status for screening of DD among children aged 1-<6 years**

| serial | **Gov.** | | **classification according to HDI** | **Hai** | **Kesm/Markaz** | **Urban** | | | **Rural** | | | | | Total |  | | | |
| --- | --- | --- | --- | --- | --- | --- | --- | --- | --- | --- | --- | --- | --- | --- | --- | --- | --- | --- |
|  |  |  |  |  |  | **Shiakha (English)** |  | **HH** | **Local Unit ( (English )** |  | **Village (English )** |  | **HH** | **HH** |  | | | |
| **1** | **Cairo** | | **High** | **AlNozhah** |  | **Al Hicksit** |  | **1390** |  |  |  |  |  | 1390 |  | | | |
|  |  |  | **middle** | **AlSaiedah Zainab** |  | **Alkabsh** |  | **1390** |  |  |  |  |  | 1390 |  | | | |
|  |  |  | **low** | **AlSharabia** |  | **Al Amiria** |  | **1390** |  |  |  |  |  | 1390 |  | | | |
| **2** | **Dakhlya** | | **High** | **AlSenbelawin** |  | **Al Sinblaween city** |  | **317** | **Kafr Alruwk** |  | **Alshalaa** |  | **905** | 1222 |  | | | |
|  |  |  | **middle** | **MietSalsil** |  | **Mit salsil city** |  | **317** | **Alatihad** |  | **AlJafara** |  | **905** | 1222 |  | | | |
|  |  |  | **low** | **AlMataria** |  | **Almataria city** |  | **317** | **Alsafra** |  | **Al Dahear** |  | **905** | 1222 |  | | | |
| **3** | **Gharbia** | | **High** | **KafrElZaiat** |  | **KafrElZaiat** |  | **260** | **Kafour Belshay** |  | **Qasta** |  | **680** | 940 |  | | | |
|  |  |  | **middle** | **Samanood** |  | **Samanood** |  | **260** | **Ziyad’s locality** |  | **Munshat Nzif** |  | **680** | 940 |  | | | |
|  |  |  | **Low** | **Markaz of Qutour** |  | **Qutour** |  | **260** | **Kotour** |  | **Khabata** |  | **680** | 940 |  | | | |
| **4** | **Fayoum** | | **High** | **Markaz of Al Fayoum** |  | **Alqism rabie** |  | **145** | **Dacia** |  | **Al Sunbat** |  | **580** | 725 |  | | | |
|  |  |  | **middle** | **Markaz of**  **Senoures** |  | **Senoures** |  | **145** | **Terrsa** |  | **Alzawia El Khadra** |  | **580** | 725 |  | | | |
|  |  |  | **Low** | **Markaz of Tamiaha** |  | **Tamiaha** |  | **145** | **Sarsna** |  | **Kafr Omira** |  | **580** | 725 |  | | | |
| **5** | **Assuit** | | **High** | **Hay Shark** |  | **Alwalidia Alwustania** |  | **235** | **Bani Hussein** |  | **Musriea** |  | **795** | 1030 |  | | | |
|  |  |  | **middle** | **Al Kousiah** |  | **Al Kousiah City** |  | **235** | **Mir** |  | **Bani Hilal** |  | **795** | 1030 |  | | | |
|  |  |  | **Low** | **Al Ghanaiem** |  | **Al Ghanaiem** |  | **235** | **Alazayiza** |  | **Al Amri** |  | **795** | 1030 |  | | | |
| **6** | **Aswan** | | **High** | **Nasr Al Nouba** |  | **Nasr Al Nouba City** |  | **280** | **Korta** |  | **Garf Hussein** |  | **410** | 690 |  | | | |
|  |  |  | **middle** | **Edfo** |  | **Al-Busaliya Bahri** |  | **280** | **Alramad Albahry** |  | **Adfu Quabli** |  | **410** | 690 |  | | | |
|  |  |  | **Low** | **Markaz KoomOmbo** |  | **KoomOmbo** |  | **280** | **Al Abbasia** |  | **Sabaa Quabli** |  | **410** | 690 |  | | | |
| **7** | **Damietta** | | **High** | **AlRawda** |  | **AlRawda** |  | **275** | **Hajaja Village** |  | **Hajaja Village** |  | **380** | 655 |  | | | |
|  |  |  | **middle** | **Al zarqaa** |  | **Alsarw** |  | **275** | **Sharmsah** |  | **Kafr Toqaa** |  | **380** | 655 |  | | | |
|  |  |  | **Low** | **Kafr-Saad** |  | **Kafer Albatiykh** |  | **275** | **Kafr Saad Country** |  | **Nawasiriya village** |  | **380** | 655 |  | | | |
| **8** | **MarsaMatrouh** | | **High** | **Marsa Matrouh** |  | **MarsaMatrouh**  **(Alsanusia & Kilo 4)** |  | **470** | **Alkasr** |  | **Alkasr** |  | **220** | 690 |  | | | |
|  |  |  | **middle** | **Al Hamam** |  | **Al Hamam City** |  | **470** | **Alsalam** |  | **Alsalam** |  | **220** | 690 |  | | | |
|  |  |  | **Low** | **AlNajyla** |  | **AlNajyla** |  | **470** | **Almathany** |  | **Almathany** |  | **220** | 690 |  | | | |
| **Total** | |  | | | | | | **10116** |  |  |  | | **11910** | **22026** |  |  |  |  |
